# Assessment and Management of Mercury Leaching from a Riverbank

**DOI:** 10.3390/toxics11020179

**Published:** 2023-02-15

**Authors:** Hasti Ziaei, Balaji Rao, Tea V. Wood, Uriel Garza-Rubalcava, Ashkan Alborzi, Huayun Zhou, Paul Bireta, Nancy Grosso, Danny Reible

**Affiliations:** 1 Department of Civil, Environmental and Construction Engineering, Texas Tech University, Lubbock, TX 79409, USA; 2 Department of Chemical Engineering, Texas Tech University, Lubbock, TX 79409, USA; 3 Department of Civil Architectural and Environmental Engineering, University of Texas at Austin, Austin, TX 78712, USA; 4 Corteva Agriscience, Indianapolis, IN 46268, USA

**Keywords:** mercury, methylmercury, drainage, inundation, DGT

## Abstract

The South River located in the city of Waynesboro, Virginia, contains mercury (Hg) contamination due to historical releases from an industrial facility operating between 1929 and 1950. In 2015, two sampling events were conducted in two of the contaminated bank regions (Constitution Park and North Park) to evaluate non-particulate total mercury (THg) and methylmercury (MeHg) concentrations in bank interstitial waters during river base flows and during bank drainage after flooding events. Porewater THg and MeHg at the bank–water interface were measured using diffusive gradient in thin-film devices (DGTs). The results showed THg mercury concentrations during bank drainage were approximately a factor of 3 higher than during base flow conditions. To have a better understanding of the parameters that control Hg leaching, a series of laboratory experiments were designed using South River sediments. The field and laboratory assessment showed that drainage/inundation cycles can lead to high THg concentration leachate from contaminated sediment due to increased partitioning from solids under oxic bank conditions and mobilization by the drainage waters. The results also demonstrated that methyl mercury concentrations at the bank–water interface are highest under base flow when conditions are more reduced due to the absence of oxic water exchange with the surface water. A remedial approach was implemented involving partial removal of surficial sediments and placement of biochar (to reduce non-particulate THg) and an armoring layer (to reduce erosion). DGT Measurements after bank stabilization showed THg decreased by a factor of ~200 and MeHg concentration by a factor of more than 20.

## 1. Introduction

The release of mercury (Hg) from industrial and mining activities [1,2] into watersheds is a global concern [3,4]. Mercury is a persistent trace element [5] and riparian sediments act as its sink. So, even after the sources of Hg are removed, historically deposited Hg in the riverbanks becomes an important continual source of Hg input into the river ecosystem [6,7]. Studies show that the potential of releasing Hg from riverbanks is significantly higher in floodplain areas [5,8,9], due to a variety of hydrological and biogeochemical mechanisms including erosion and Hg release and leaching in the dynamic floodplain environment [8,9]. Hg release and leaching is particularly important since this is considered to be the most bioavailable form of Hg [10,11]. In the dissolved form, Hg (primarily Hg^2+^) may be complexed with inorganic and organic components [4,12] and transformed to methylmercury by methylating bacteria [3]. Methylmercury is a neurotoxin compound that can enter the food chain by bioaccumulation and biomagnification in fish tissue and cause serious health problems for human [4,13,14].

The bank of the South River in Waynesboro, Virginia contains mercury (Hg) contamination due to historical releases of mercuric sulfate. Hg concentrations in the South River floodplain have been observed to be highest in areas with 2–5 years of flood frequency [15]. Some reports have indicated relatively low concentrations of Hg in the groundwater and bank interstitial waters (e.g., 2.9 ng/L Hg by Eggleston [9]) and as a result, bank erosion has been identified as more important than leaching as a release back to the river [16,17]. These same studies suggest that 78−98% of mercury released as a result of erosion is particulate-bound Hg. Although particulate-bound Hg may dominate the total release from the banks, releases of the more available dissolved Hg [3,18] may dominate methylation. Hg in the bank interstitial waters may be mobilized by both net groundwater movement as well as cyclic inundation and drainage as a result of fluctuations of the river level [19,20].

During flooding events, the water level in the South River in the vicinity of Waynesboro can increase 3–5 ft inundating the banks with water which drains back to the river after the water returns to normal levels. This cycle can facilitate non-particle associated Hg back to the river [21]. Kelly et al. showed that inundation and drainage conditions can increase the mobility of metals which positively correlated with dissolved organic matter (DOM) concentration [22]. Changes in pH and redox during inundation–drainage cycles increases DOM release, promoting the formation and release of organo-Hg complexes in porewater [22], In addition, the change of redox conditions during inundation of oxic river waters during high flood stage can facilitate oxidation of precipitated Hg forms as well as mobilizing mercury in water bound by capillarity in the unsaturated zone.

**Figure 1:**
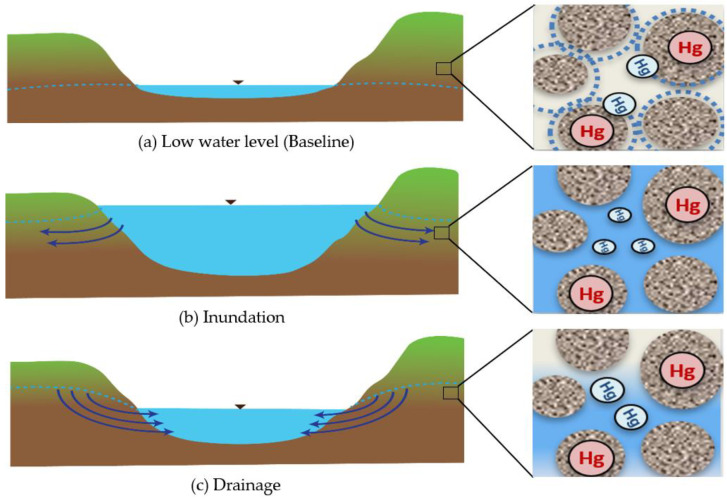
The conceptual model: (**a**) Hg contaminated porewater is not moving because of capillary suction in the unsaturated soil; (**b**) Aerated water inundates the bank and the Hg in porewater can exchange with water; (**c**) Hg Contaminated porewater slowly drains back to the river as the water level recedes.

This study is based on the hypothesis that drainage and inundation as a result of storm events, tides, and seasonal changes can affect Hg mobility and release from contaminated banks. During base flow conditions when the water level is low, Hg contaminated porewater in the oxic, unsaturated portion of the bank is largely immobile due to capillarity. Herein, we will refer to water levels and mercury exchange at base flow conditions as baseline conditions. Deeper in the bank in the saturated zone, anoxic conditions prevail and Hg methylation is likely to occur (Figure 1a). Then, during a storm event giving rise to increases in water level, aerated water inundates the bank and the Hg in previously capillary bound porewater exchanges with mobile water. Hg methylation is also likely to decrease (Figure 1b) due to the inundation of oxic water in both the unsaturated and previously saturated zone. As the water level recedes in the days following inundation, contaminated porewater drains back to the surface water, leading to elevated THg and potentially decreased MeHg concentrations in the water (Figure 1c). In this paper we examine these mechanisms in the South River. We expect that the mechanisms of those processes are common to mercury contaminated riverbanks in other locales as a result of storm events or seasonal or tidal fluctuations in water level.

Porewater for THg concentration was sampled for 6 years (2015 through 2020) in both baseline and during drainage conditions in the aftermath of storm events. In 2016, some of the bank surface soils were removed and replaced with biochar and an armoring layer. These were implemented for both bank stabilization and to reduce particle- and non-particle-related release of mercury [15,23,24]. Monitoring was conducted both before and after bank stabilization, providing insight into the ability to control non-particulate mercury release from the banks.

We also examined the physiochemical factors that govern the porewater concentration of MeHg in the bank sediments. Mercury methylation usually occurs near the anoxic part of the sediment–water interface [25] and is mediated by bacteria, primarily sulfate-reducing bacteria (SRB) and iron-reducing bacteria (IRB) [4,25,26,27]. MeHg production depends on sediment geochemistry, Hg speciation, and bacterial uptake and activity [3,28]. Sediment geochemistry as its role in controlling Hg speciation, transformation, and mobility [8], was assessed through variety of ancillary measurements such as anions (sulfate, chloride, sulfide, etc.), redox, dissolved oxygen, and temperature with Hg methylation.

## 2. Materials and Methods

### 2.1. Study Area

**Figure 2:**
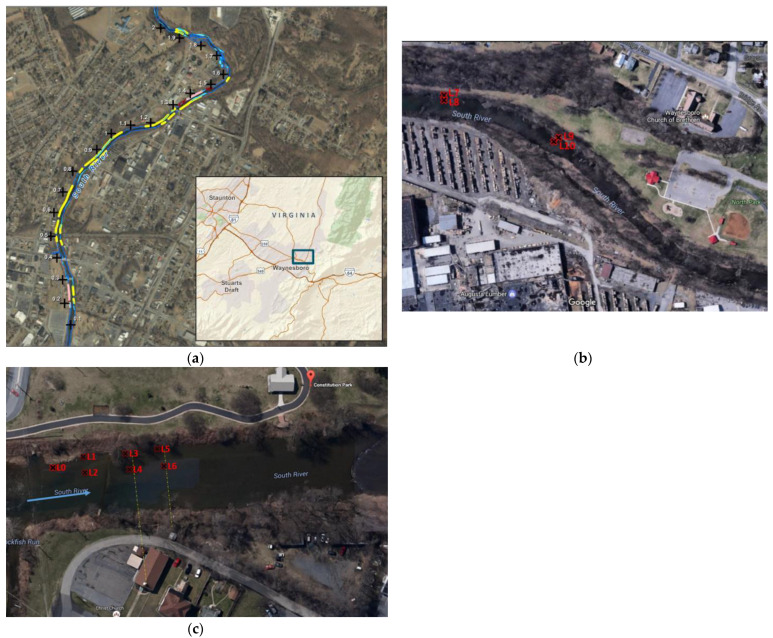
The area of study: (**a**) South River, near Waynesboro, VA; (**b**) Constitution Park and the sampling locations in bank (L0, L1, L3, L5), and channel (L2, L4, L6) that were used for sampling (2015–2020); (**c**) North Park and the sampling locations in riverbank (L7 and L9) and channel (L8 and L10) that were used for sampling in 2015.

The area of study (Figure 2a), includes North Park, 0.96–1.00 miles (Figure 2b), and Constitution Park, 0.18–0.21 miles downriver of the source location (Figure 2c) in Waynesboro, VA. As a result of the historical Hg discharge, significantly elevated Hg concentrations have been measured ranging from 0.16 to 3610 mg/kg at Constitution Park, and from 0.04 to 291 mg/kg at North Park [24]. In 2015, sampling was conducted at 3 locations of Constitution Park (bank (L1, L3, L5) and adjacent channel locations (L2, L4, L6)) as well as four locations at North Park (bank (L7 and L9) and the adjacent channel (L8 and L10)). Bank locations contain fine grained sediment at the surface followed by significant amounts of sand and gravel 1–2 ft below the surface. Channel locations were primarily characterized by sand and gravel. During sampling, near surface concentration profiles of THg and MeHg were analyzed at the bank water interface where seepage from the bank would be expected to be highest. Location numbers increase in the downstream direction as does the bank soil mercury concentration over this stretch of river. The current discussion focuses on location L5 at Constitution Park and L7 at North Park, because of the higher concentration of Hg in these locations. At Constitution Park, the bank was stabilized in 2016 by removal of a portion of the contaminated soil and placement of a 6-inch thick geocell containing sand and biochar to control Hg leaching and a 6–12 inch armoring layer of gravel and rock to control erosion [24]. In total, 200 cubic yards of the hazardous bank soil and 100 cubic yards of non-hazardous bank were removed, but even after the soil removal, an average of 150 mg/kg Hg remained in the near surface soils of L5 which had an average concentration of 769 mg/kg before the bank stabilization.

Separate laboratory experiments suggested that the biochar would exhibit a Hg partition coefficient of at least 1000 L/kg [19] and was used to offset the lack of significant sorption capacity in the armoring rock. A 12-inch-thick layer of planting substrate with biodegradable coir fabric was placed on the upper bank to protect the area from erosion until the vegetation could regrow. Porewater monitoring was conducted before and after the bank stabilization efforts. No stabilization was attempted at North Park and sampling at that location was only conducted prior to 2016. 

### 2.2. Passive Samplers and Instrumentations

#### 2.2.1. DGT Samplers: THg and MeHg Analysis

Porewater THg and MeHg concentration was determined with diffusion gradient in thin film devices (DGTs) in order to allow in situ measurement with minimal disturbance of redox conditions [23,29,30,31]. Ref. [18] showed that DGTs largely measure non-NOM- and non-particulate-related Hg since colloidal and particulate-bound Hg diffuse extremely slowly through the DGT membrane layers. DGT profilers were used to evaluate the surficial 15 cm of sediment with 2 cm resolution. DGT piston samplers, which can measure THg and MeHg at a point, were used at the sediment surface and in the overlying water. DGTs were fabricated in-house, and they consist of three layers. The resin gel (sorbing layer) was made using 3-mercaptopropyl functionalized silica gel (3MFSG) Si Thiol resin from Biotage (Uppsala, Sweden) immersed in agarose gel from Thermo Fisher Scientific (Waltham, MA, USA). The diffusive gel was also made of agarose and covered by a 0.45-micron filter from Sigma-Aldrich (St. Louis, MO, USA) to protect the resin gel. Following the assembly of DGTs, samples were de-aerated in sodium nitrate with ultra-pure N_2_, and profilers were bagged inside of anaerobic chamber and placed on ice until site deployment (DGT fabrication details can be found in [18]). 

Three DGT profilers were deployed at each of the locations in soft sediment separated by approximately 10 cm, and six DGT pistons were used for two of the most contaminated locations (3 and 5) to measure the THg and MeHg in the water column. After 48 h, samplers were retrieved, rinsed with deionized (DI) water to remove soil particles from piston and profiler bodies and double bagged, placed on ice and returned to the lab for processing. During processing, the first two layers of filter and diffusive layer were removed and the sorbing layer was split in half (for separate THg and MeHg analysis). A single sample for both THg and MeHg was generated from the piston DGTs while the profilers were sectioned into 2 cm increments referenced to the sediment–water interface (0 cm).

Total mercury resin gels were eluted using 3 mL of concentrated trace-metal grade hydrochloric acid for 24 h and analyzed on a Brooks Rand Merx-T cold vapor atomic fluorescence spectroscopy (CVAFS) using the EPA Method 1631 [32]. The extraction recovery of THg from DGT was 97 ± 6% [18]. Resin gels for MeHg analysis were eluted in 10 mL of 30% nitric acid and digested overnight. The MeHg recovery by this extraction approach was 91 ± 9% [23]. The samples were analyzed by Brooks Rand Merx-M cold vapor atomic fluorescence spectroscopy (CVAFS) using EPA Method 1630 [33]. 

#### 2.2.2. Diffusion Samplers: Anions and velocity Measurement

##### Measurement of Inorganic Anions in Pore Water

In 2017, 2018, and 2020, inorganic anions in pore water were analyzed using equilibrium diffusion sampling [34,35,36]. The sampling device was made of polycarbonate and rectangular 60.5 × 10.3 × 2.4 cm^3^ with 37 cells of dimensions 1 × 6 × 0.5 cm^3^ spaced 0.5 cm apart. The cells are filled with distilled water spiked with potassium bromide (~100 mg/L of Br^−^) and covered by a 0.45 µm Nylon membrane (Sterlitech, Kent, WA, USA) and a 10 µm Nylon mesh (Component Supply, Sparta, TN, USA) for protection of the membrane. The membrane/mesh layers were held in place with an acrylic cover plate and stainless-steel screws. Once the sampler was deployed, dissolved species diffuse across the membrane into the cells. Water samples were collected from the reservoirs via a syringe and filtered (0.45 µm) before direct analysis by ion chromatography. 

Br^−^ diffuses out of the sampler and the measured loss is used to calculate the extent of equilibration of each cell. For cells not fully equilibrated at the end of the sampling period (i.e., those containing measurable Br^−^ at retrieval of sampler), the captured concentration of each inorganic ion is corrected according to equation

$$C_{pw}=\frac{C_{i}}{1-C_{Br}/C_{0}}$$

where $C_{pw}$ is the estimated pore water concentration of targeted species, $C_{i}$ is the concentration of targeted species at the end of the sampling period, and $C_{Br}$ and $C_{0}$ are the final and initial concentration of Br^−^ [37].

##### Estimation of Porewater Velocity

Estimates of pore water velocity were also obtained from a diffusion sampler in 2018 and 2020 using the method of Schneider et al. (2019) [38]. The methodology was not available in earlier sampling events. In short, the Br^−^ release from 4 cells with different area to volume ratios are used to estimate an effective mass transfer coefficient at the membrane–water interface which has been correlated with interstitial water velocity in sediments. The sampling device was composed of a polycarbonate rod (diameter of 6.4 cm and length of 52.9 cm) in which there were three sets of velocity cells. The cells were filled with distilled water spiked with potassium bromide (~100 mg/L of Br^−^) and covered by a 0.45 µm Nylon membrane (Sterlitech, Kent, WA, USA), a 10 µm Nylon mesh (Component Supply, Sparta, TN, USA), and a polycarbonate cover plate held in place with stainless-steel screws. The ratio of the mass transfer coefficient at the surface of the cell and the ratio of the Br^−^ concentration at any time to the initial concentration is given by mass balance

$$\frac{C_{t}}{C_{0}}=\exp\left\{-\frac{kA}{V}t\right\}$$

where $C_{t}$ and $C_{0}$ are the final and initial concentrations of Br^−^, k is the overall mass-transfer coefficient between sampling cell and sediment pore water, A is the available area for mass transfer, V is the cell volume, and t is the sampling period. Because of the different $\frac{A}{V}\mathrm{ratios}$, four different $C_{t}/C_{0}$ values are obtained from each set of velocity cells at retrieval. These values are used in the equation above to obtain a representative mass-transfer coefficient for bromide, k, through non-linear regression. The value of k can be used to estimate average pore water velocity, u, using membrane properties and the laboratory derived relationship presented in [38].

#### 2.2.3. Deployment and Retrieval of Diffusion Samplers

For deployment, pre-cleaned (soap, water, and distilled water) equilibrium and velocity diffusion samplers were prepared in the field. Assembly was conducted while the devices and components (membrane, mesh, cover plates, and screws) were fully submerged in a solution of distilled water and potassium bromide (~100 mg/L of Br^−^) after releasing any trapped air bubbles. Cells were covered with the membrane and the mesh, and materials were secured with cover plates and stainless-steel screws. The devices were deployed in the sediments at the bank–water interface by hand.

At the end of the sampling period, the samplers were retrieved and sampled immediately. The surface of each sampler was rinsed with distilled water to remove sediments and to expose the cells, and water samples were extracted with a sterile plastic syringe and needle. Samples were placed in 15 mL polypropylene vials and shipped on ice to Texas Tech University (Lubbock, TX, USA) for analysis of inorganic anions (Cl^−^, Br^−^, NO_3_^−^, and SO_4_^2−^) by ion chromatography according to EPA method 300.1 (see Table S1 in SI for the number of samplers and deployments period of each sampling).

### 2.3. Dissolved Oxygen, Redox, PH and Sulfide

An ion specific electrode system using a Unisense Microsensor Multimeter (Version 2.01) coupled with OX-N sensor for oxygen measurement, H_2_S-NP sensor for measuring H_2_S gas, pH-NP, RD-N and REF-RM electrodes for measuring pH and redox, respectively. The Unisense system was used to measure depth profiles of oxygen and sulfide, pH, and redox potential in the interstitial water with 1 cm resolution. Probes were calibrated in the lab before and after field measurements. The electrodes were advanced until refusal or until maximum depth was reached (8 cm). Probes were rinsed with deionized water (DI) water in between the location deployments. 

### 2.4. Laboratory Experiments

A series of experiments was designed to study the parameters that control Hg leaching from the South River sediments under more controlled conditions in the laboratory. Selective sequential extraction, bank sediment mesocosm, and a bank sediment column study were conducted. 

Selective sequential extraction was used to characterize the Hg associations on the solid and indicate the ease of extraction and leaching from the bank sediment. For this purpose, five extraction solutions from low to high strength were added to the bank sediment including DI water which can extract water soluble compounds (F1), 0.01 M HCl + 0.1 M CH_3_COOH which is a weak acid (F2), 1 M KOH for extracting organo chelated materials (F3), 12 M HNO_3_ used for extracting elemental mercury (F4), and aqua regia which can extract mercuric sulfate (F5) [39]. Each extracted solution was analyzed on a Perkin Elmer Elan DRC-e ICP-MS and any samples lower than 1 µg/L on the ICP were reanalyzed on a Brooks Rand Merx-T CVAFS by EPA Method 1631. 

**Figure 3:**
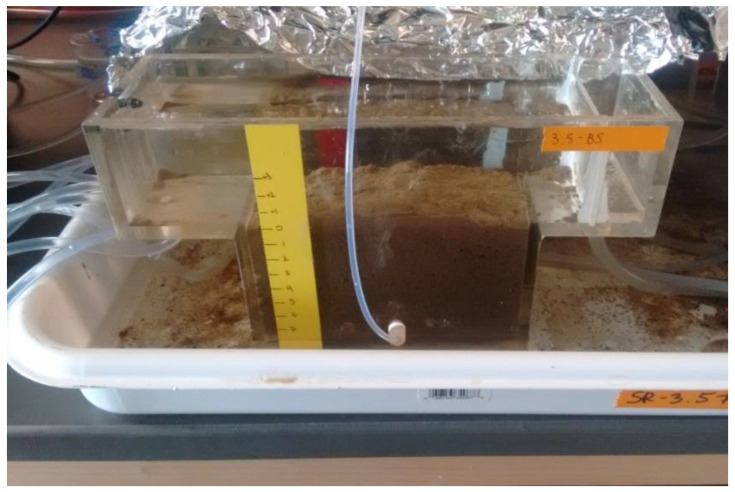
South River RRM3.5 Bank Sediment Mesocosm.

A bank sediment mesocosm was designed to simulate the drainage and low flow baseline conditions (minimal bank exchange). To simulate baseline conditions, homogenized sediment was placed into acrylic T-cells (Figure 3) with surface dimensions of 5 cm × 15 cm and depth of 8 cm. On top of the sediment, a 5 cm freshwater column that consisted of 0.4 mM calcium chloride, 0.5 mM sodium chloride, 0.5 mM potassium chloride, and 0.2 mM sodium bicarbonate was circulated over the bank soil with a residence time of 1.25 exchanges per week. The mesocosm was given enough time (4 weeks) to reach established steady redox conditions as indicated by voltammetry (SI). Then, the porewater THg concentration were measured using in situ DGT piston samplers. After DGT retrieval, water from the mesocosms was drained and used for filtered and unfiltered THg concentration. Filtration was performed using a 0.45-µm polyethersulfone filter. Samples were preserved in 1% bromine mono chloride and then analyzed on a Brooks Rand Merx-T analyzer. The approximate porewater volume of the mesocosm was 240 mL. Porewater measurements were limited to the first 100 mL drained in order to minimize dilution with overlying water.

After sampling the baseline conditions, oxygenated freshwater was pumped through the bank sediment for 15 days with a seepage rate of 0.05 cm/min to mimic inundation conditions. Then, water pumping was stopped and the oxic water was allowed to equilibrate with the bank soil for 3 days. The system was allowed to drain then filtered and unfiltered water were used for THg measurement. The dissolved oxygen profile with depth was measured using voltammetry. 

A final set of experiments was conducted in a column to evaluate THg release as a result of the transition from anaerobic (baseline) to aerobic (inundation) and back to anaerobic conditions (baseline). To simulate baseline conditions, de-aerated freshwater was pumped into a homogeneously packed sediment column and allowed to equilibrate for two weeks until THg concentration in the outlet had stabilized. After establishing steady conditions, aerated water was pumped into the column and the outlet water was tested over 4 days (~15 pore volumes) for the target parameters. After 4 days, feedwater was switched back to de-aerated water and the outlet was sampled for an additional 5 days (~20 pore volumes). Effluent conditions were steady within 2 pore volumes after each transition. 

The column effluent was monitored for THg, sulfide, total iron, total manganese (Mn), DOC, and pH. pH and sulfide were measured directly in the column effluent while metals, anions, and DOC were measured after filtration with 0.45 µm polyethersulfone filter to be used for metals, anions, and DOC analysis. THg samples were preserved in 1% bromine monochloride and stored at 4 °C until analysis according to EPA 1631 on a Brooks Rand Merx-T system. Samples for ICP were preserved with 2% trace-metal grade nitric acid and stored at 4 °C until analysis. ICP samples were run on a Perkin Elmer Elan DRC-e ICP-MS according to EPA Method 200.7. Samples for anions analysis were also stored at 4 °C until analysis on a Dionex IC system with an IS25 Isocratic pump, a CD20 conductivity detector, and an AS40 autosampler. In order to measure the DOC of the samples after preserving the samples with 0.2% trace metal grade hydrochloric acid and storing at 4 °C, they were analyzed on a Vario TOC Select (Elementar, Germany) according to EPA Method 415.3, Revision 1.1.

## 3. Results and Discussions

### 3.1. Drainage vs. Baseline Conditions

#### 3.1.1. Field Results

**Figure 4:**
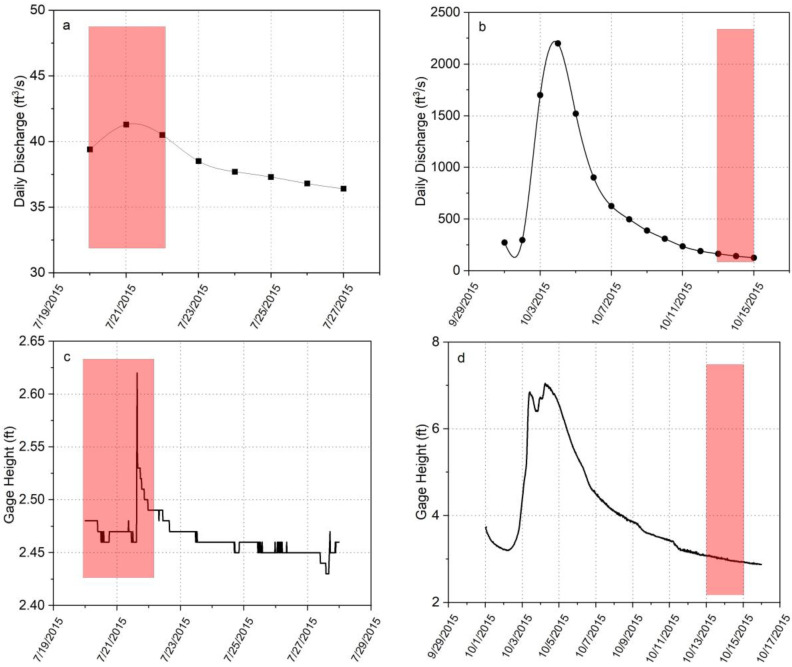
USGS stream flow and height data for South River in 2015: (**a**) stream flow during baseline condition (July 2015), (**b**) stre.am flow during drainage condition (October 2015), (**c**) stream height during baseline condition (July 2015), (**d**) stream height during drainage condition (October 2015).

**Figure 5:**
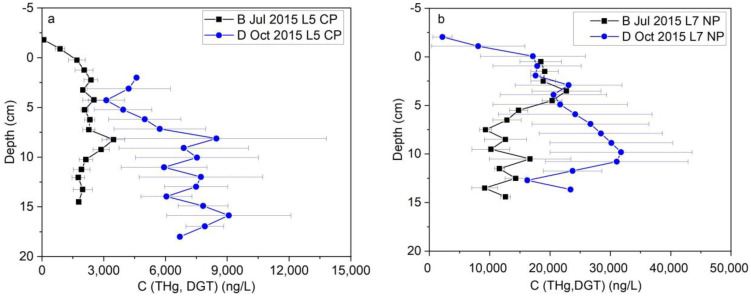
(**a**) Porewater THg concentration measured with DGT at L5 (Constitution Park) during Baseline (B) (July 2015) and Drainage (D) (October 2015); (**b**) porewater THg concentration measured with DGT at L7 (North Park) during Baseline (B) (July 2015) and Drainage (D) (October 2015); on the y-axis, 0 represents the water/sediment interface and the negative numbers refer to distances above the interface (i.e., in the water column).

Two field sampling efforts were conducted in 2015 before any bank stabilization efforts to assess Hg concentration in porewater under both drainage and low flow baseline conditions in both Constitution Park and North Park. The first sampling was performed in July 2015 during a baseline condition when the river flowrate was ~40 cubic feet per second (cfs), very close to the historical median flow of ~46 cfs. (Figure 4a), and the average water level was 2.55 ft (Figure 4b). The water temperature was 26 °C. Samples were also collected in October 2015 during the period immediately following a storm event resulting in a peak flow of ~2000 cfs (Figure 4c) and water level increased to 7 ft (Figure 4d). The river water temperature during the sampling was 16 °C. Porewater THg concentration in Constitution Park and in the most contaminated location, L5, during the baseline condition was 2.2 ± 0.5 µg/L, which increased by a factor of ~3 to 6 ± 2 µg/L during the drainage condition in October (Figure 5a). At North Park, the porewater THg concentration during the baseline condition was 15 ± 4 µg/L, which increased to 21 ± 8 µg/L during the drainage condition in October (Figure 5b). Although the depth-averaged concentration changed only modestly, this is because the concentration in the layer likely to remain oxic under both conditions (the upper 5 cm of sediments) were largely unchanged. The concentrations below a 5 cm depth increased from approximately 10 µg/L under baseline conditions to more than 30 µg/L during drainage conditions, again a factor of 3. We hypothesize that Hg in the unsaturated portion of the bank is largely held in immobile water trapped by capillary forces and equilibrates with the adjacent contaminated soil. During inundation and drainage, this water is mobilized and can drain back into the river. 

Despite the much higher THg concentrations during the drainage conditions, MeHg in the porewater decreased from 20–40 ng/L during the baseline to <5 ng/L during the drainage conditions at Constitution Park. At North Park, porewater MeHg concentration was much higher than Constitution Park, but it also decreased from ~70 ng/L to ~18 ng/L in average from baseline to drainage condition. This is likely due both to the aerobic conditions resulting from the inundation of oxic river water and the cooler temperatures reducing microbial activity. Thus, THg leaching from a bank during drainage when compared to the baseline conditions was repeated in other sampling events and at other locations along the South River. Typically, THg concentrations increased 2–3 times during the drainage while MeHg stayed the same or decreased during drainage measurements. 

During baseline conditions, the sediments are largely reduced below the water table and bank water is expected to be exchanging only slowly with the river water. During a high flow event, aerated river water inundates the bank and then slowly drains back to the river (over ~1 week) as the river level recedes. 

#### 3.1.2. Laboratory Results

The results of selective sequential extraction, laboratory mesocosms and column studies were used to better understand the increase in Hg leaching during drainage conditions. Based on the results, F1, F2, and F3 solutions extracted 0.5%, 1.9%, and 5.5% of the mercury in the solid phase. We expect that the F1 and F2 fractions are readily available and that the F3 fraction is likely to partition into the porewater during oxygenated conditions occurring during inundation or held in capillary bound water in the unsaturated zone. The F3 fraction is 3–15 times greater than the F1/F2 fractions, consistent with the observed increases in THg in water draining from banks after high flow events in the field. The results also suggest, however, that the bulk of the Hg (>90%) is tied up in largely immobile precipitated phases. 

**Figure 6:**
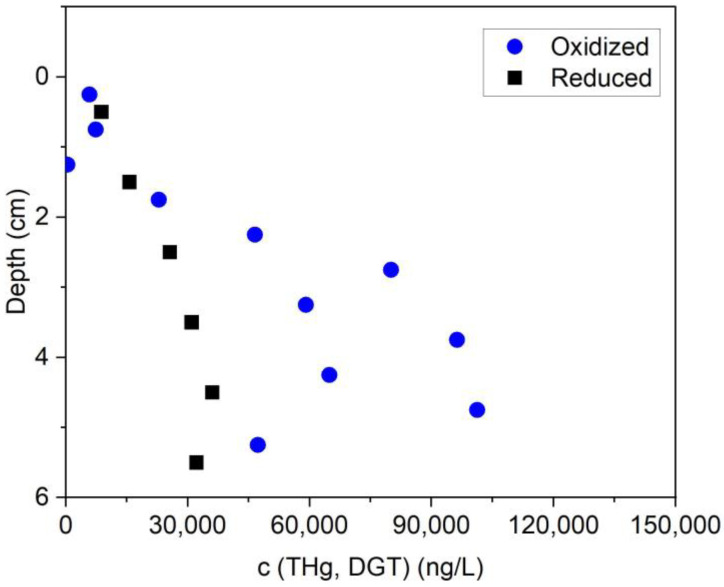
Porewater THg concentration in lab mesocosm using Sediment from South Riverbank RRM 3.5.

The mesocosm THg results are shown in (Figure 6). Oxygen decreased to 0 within 2 cm of the surface of the mesocosm and conditions were anoxic throughout most of the mesocosm sediments (Figure S1). THg concentration in the anoxic sediment porewaters was between 25 and 36 µg/L. After the introduction of oxic water simulating inundation, the THg concentration in the interstitial water increased to 45–100 µg/L or 2–3 times greater. 

The column experiments showed a stable THg concentration in effluent water under reduced conditions of 10 µg/L. Switching to an oxic feedwater to simulate bank inundation, the THg concentration increased to 35 µg/L, or approximately 3 times higher. pH also decreased to 6.85–7.52 from the initial value of 7.54–7.84 (Figure S2). The lower pH during the oxygenated condition can be due to a variety of parameters and reactions such as oxidation of organic matter, sulfide, and iron compounds [40], processes that parallel the oxidation of the Hg precipitants in the F3 fraction. In this experiment, sulfide was under the detection limit of 0.5 µmol/L at any time. Additionally, total iron and DOC did not change significantly during oxidized and reduced conditions. However, total Mn showed to be higher under reduced condition (Figure S3). Increasing in Mn^2+^ which is soluble is considered as an indication of a reduced condition. Under a reduced condition, Mn is more mobilized, and oxidation of system decreases the Mn concentration in porewater [41].

### 3.2. Bank Stabilization Effect on THg

**Figure 7:**
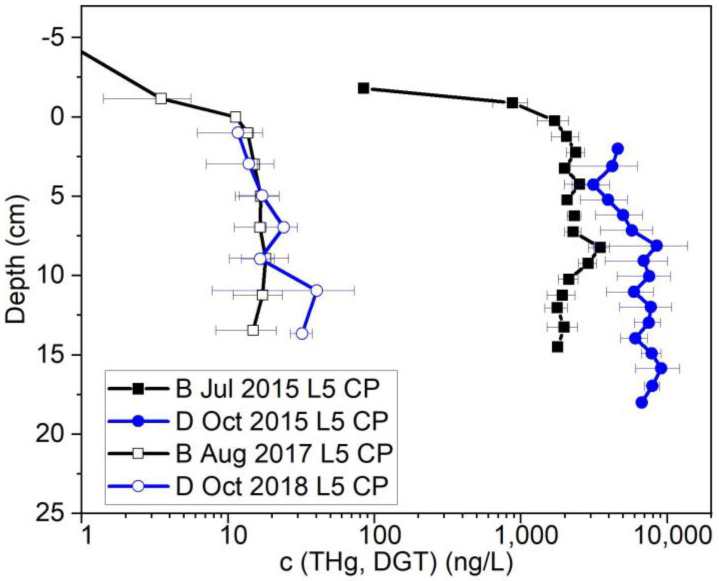
Porewater THg concentration measured by DGT in L5 at Constitution Park during different sampling events both before and after bank stabilization under baseline (B) and drainage conditions (D); on the y-axis, 0 represents the water/sediment interface and the negative numbers refer to distances above the interface (i.e., in the water column).

The bank stabilization and biochar placement that was conducted in 2016 was evaluated to determine if it had a significant effect on THg release. Bank stabilization efforts dramatically reduced THg leaching from the bank in all locations (Table S2), especially L5 at Constitution Park with a maximum THg concentration of 9070 ng/L in October 2015 that was reduced to less than 50 ng/L after bank stabilization (Figure 7). After the bank stabilization, the porewater concentration of all locations is comparable with the reference location L0 which exhibited low contamination levels, suggesting that the bank stabilization effort was effective (Table S2). 

### 3.3. MeHg Behavior after Bank Stabilization

**Figure 8:**
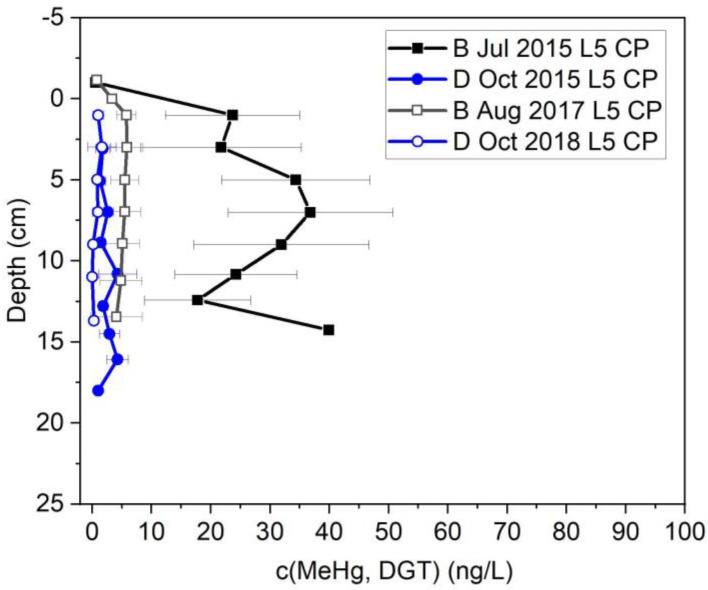
Porewater MeHg concentration measured by DGT in L5 at Constitution Park during sampling events before and after bank stabilization and during baseline (B) and drainage (D); on the y-axis, 0 represents the water/sediment interface and the negative numbers refer to distances above the interface (i.e., in the water column).

Figure 8 shows MeHg concentration profile in location L5. Unlike THg, MeHg was not affected substantially by bank stabilization at most locations. In addition to the availability of dissolved Hg, methylation occurs under specific biogeochemical conditions that require many factors including temperature, reduced conditions, the presence of methylating bacteria, and availability of food sources or electron donors for bacteria. MeHg concentration in most locations was similar before and after bank stabilization. At location L5, where the THg decreased dramatically by stabilization, MeHg was reduced by an order of magnitude under baseline conditions from a maximum 40 ng/L in July 2015 to 5.8 ng/L in Aug 2017. MeHg concentration during drainage conditions before and after the bank stabilization remained low, 2.4 ± 1.2 ng/L in October 2015 and 0.7 ± 0.6 ng/L in October 2018.

**Figure 9:**
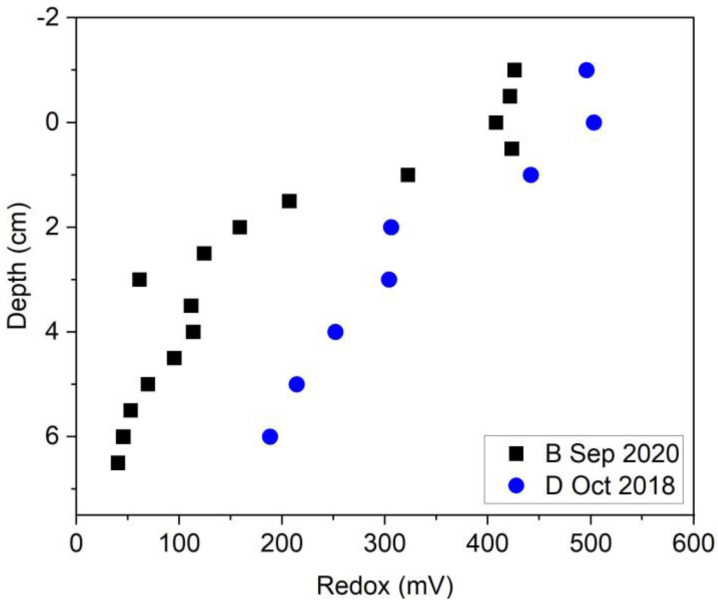
Redox potential in L5 at Constitution Park (2018 and 2020); on the y-axis, 0 represents the water/sediment interface and the negative numbers refer to distances above the interface (i.e., in the water column).

The hypothesis that fewer reducing conditions are present during drainage of oxic water back to the river was tested by measurements of oxidation-reduction potential. Figure 9 shows more reduced conditions during baseline (2020) than during the drainage conditions (2018). The average MeHg concentration in L5 during the baseline condition of 2017 and 2020 was 5 ± 2 ng/L and 5 ± 4 ng/L, respectively, whereas the MeHg concentration during drainage condition of 2018 was 0.7 ± 0.6 ng/L. Higher methylation under baseline conditions relative to drainage conditions was also measured prior to bank stabilization (27 ± 12 ng/L vs. 2 ± 1 ng/L). Inundation and drainage condition introduce oxygen to the surficial sediment which is not favorable for Hg methylators. Generally, Hg methylation takes place under anaerobic condition where the anaerobic bacteria such as SRB and IRB are active. So, oxygen disturbs productivity and activity of those bacteria. Additionally, temperature is another factor that has an effect on methylation by increasing bacterial activity; therefore, the results show that the higher methylation occurs under a reduced and warmer condition.

**Figure 10:**
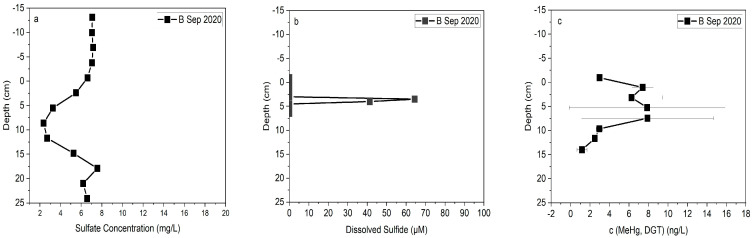
(**a**) Sulfate concentration, (**b**) dissolved sulfide concentration, and (**c**) MeHg concentration, profiles in L5 at Constitution Park (2020); on the y-axis, 0 represents the water/sediment interface and the negative numbers refer to distances above the interface (i.e., in the water column).

The reducing conditions indicated by the oxidation-reduction potential were also supported by sulfide measurements. No sulfide was detected during the inundation/drainage conditions of 2018 where the reduced condition was (>100 mV) in all depths above 6 cm. However, >60 µM sulfide was detected during the sampling effort of 2020 under baseline conditions and reduced conditions (<100 mV) were detected in all locations at a bank soil depth of 2.5–3 cm (Figure 10b, Figures S4 and S5). The sulfate concentration with the constant concentration of 7 mg/L in surface water was gradually depleted to 2 mg/L from below the interface to 10 cm in depth and then it started to increase to 15 mg/L (Figure 10a). At the same time, the methylmercury profile started to increase from right below the interface, and it reached the maximum level (7.6 ng/L) in the depth of 3–5 cm, then decreased to near 1 ng/L in a depth of 10 cm and remained constant (Figure 10c). This showed activity of sulfate reducing bacteria in this condition. 

### 3.4. Flux of Mercury at Sediment-Water Interface

Table S3 shows the estimates of pore water velocity in sediments from equilibrium diffusion samplers. Observations from the two sampling events show a stronger mass exchange at the sampler–sediment interface in locations L0 and L5 if compared to L1 and L3. Consistency in those observations suggests focused discharge points with high interstitial velocity. In the case of L5, where the faster loss of tracer was measured, results indicate that seepage occurs mainly in the bank, since the sampler deployed in the riverbed (1.4 m from shore) exhibited a smaller rate of mass exchange, which translates to a small pore water velocity.

The average interstitial velocity from diffusion samplers can be used to estimate advective fluxes of species from the bank. At the bank of location L5, where higher seepage velocity and THg concentrations was observed, a geometric mean of Darcy’s flux is ~17 cm/d. Thus, an estimate of mercury’s flux during drainage conditions per foot of bank length is

$$\left(∼17\;\mathrm{cm}/d\right)\left(30.7\;\mathrm{ng}/L\right)\left(∼20\;\mathrm{cm}\right)\left(10^{-3}\;L/{\mathrm{cm}}^{3}\right)\left(30.48\;\mathrm{cm}/\mathrm{ft}\right)≅320\frac{\mathrm{ng}}{\mathrm{ft}\cdot d}$$

which assumes that the top ~20 cm of the sediments is the main section of the bank contributes to the advective flux of mercury since observations indicate a significant drop in pore water velocity below that depth.

Assuming drainage velocities are similar before and after bank stabilization (no low permeability layer was placed during stabilization), a similar flux estimate of total mercury for drainage conditions prior to bank stabilization at L5 is 65,900 ng/ft/d, which is ~200 times higher than current fluxes during similar conditions.

## 4. Conclusions

The field and laboratory assessments of THg leaching from sediment into porewater showed that drainage/inundation conditions can lead to a higher release of non-particulate Hg which is the result of both a higher exchange between the bank and river as well as introducing aerated water into the bank that tends to decrease Hg partitioning into immobile solid phases. The results also demonstrated that reduced condition and higher temperature are two effective factors that govern Hg methylation. Hg methylators such as IRB and SRB are active under an anaerobic condition and temperature increases their activity.

Assessment of bank management effort indicated that capping effectively reduced THg concentration by a factor of more than ~200 and decreased MeHg concentration by a factor of more than 20. 

## Data Availability

Data are included in the SI and the remainder is available from the corresponding author.

## Supplementary Materials

The following supporting information can be downloaded at: https://www.mdpi.com/article/10.3390/toxics11020179/s1, Table S1: Equilibrium and velocity samplers’ locations and deployment periods, Figure S1: Depth profile of Oxygen concentration from the lab mesocosm using South River sediment (RRM3.5) under Oxidized and Reduced Conditions, error bars represent standard deviation from 3 replicates, Figure S2: pH of the column effluent per pore volumes using South River sediment from RRM3.5 under oxidized and reduced conditions, Figure S3: Total Manganese of the column effluent per pore volumes using South River sediment from RRM3.5 under oxidized and reduced conditions, Table S2: Average THg concentration ± one standard of deviation (*n* = 3) in all bank locations of Constitution Park before and after the bank management, Figure S4: Redox Potential during Drainage condition in 2018 in bank locations of Constitution Park, Figure S5: Redox Potential during baseline condition in 2020 in bank locations of Constitution Park. The location 3 and location 5 were both rocky so the point that was selected was between these two locations, Table S3: Bromide mass-transfer coefficients and estimates of interstitial water velocity from equilibrium diffusion samplers.
