# Supplementary material for: Assessment and Management of Mercury Leaching from a Riverbank
Source: Toxics. 2023 Feb 15;11(2):179. doi: 10.3390/toxics11020179 (PMC9962584; doi:10.3390/toxics11020179)
Supplement: Supplementary file 1 [file toxics-11-00179-s001.zip › toxics-2193303-supplementary.pdf]

toxics MDPI

Supplementary Materials

Assessment and Management of Mercury Leaching from a Riverbank

Hasti Ziaei [1], Balaji Rao [1], Tea V. Wood [1], Uriel Garza-Rubalcava [2], Ashkan Alborzi [1], Huayun Zhou [1], Paul Bireta [3], Nancy Grosso [4] and Danny Reible [1,2,*]

Table S1. Equilibrium and velocity samplers' locations and deployment periods

Sampling Events	Equilibrium Samplers		Velocity Samplers	
	locations	Deployment Period (days)	locations	Deployment Period (days)
2017	L0, L1, L3 and L5	4	-	-
2018	L0, L1, L3 and L5	3	L0, L1, L3 and L5	3
2020	L0, L1, L3 and L5	2	L0, L1, L3 and L5	5

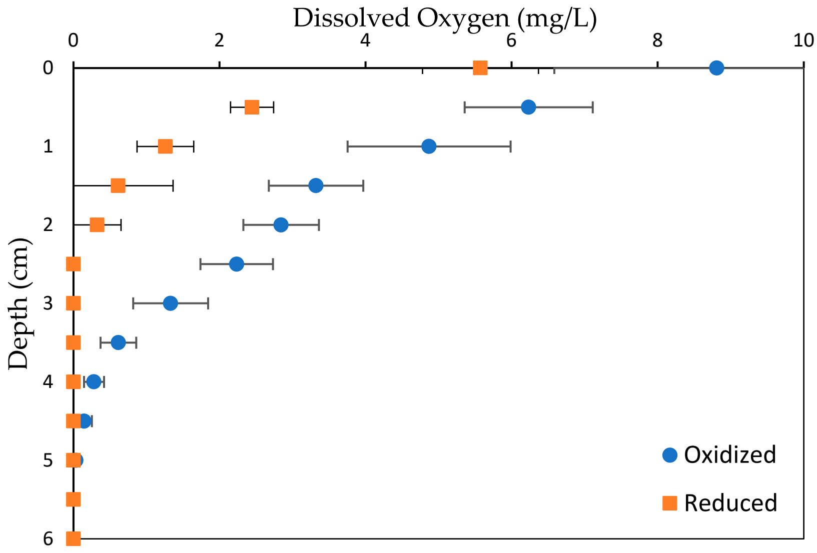

Figure S1. Depth profile of Oxygen concentration from the lab mesocosm using South River sediment (RRM3.5) under Oxidized and Reduced Conditions, error bars represent standard deviation from 3 replicates

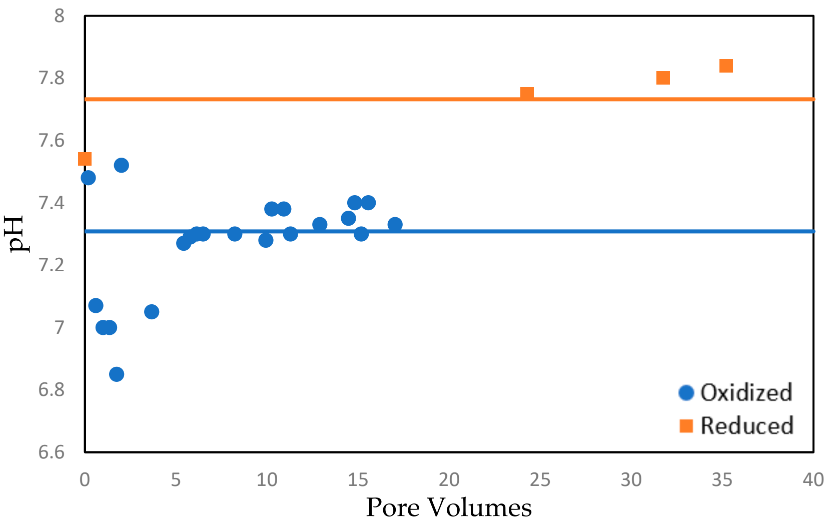

Figure S2. pH of the column effluent per pore volumes using South River sediment from RRM3.5 under oxidized and reduced conditions.

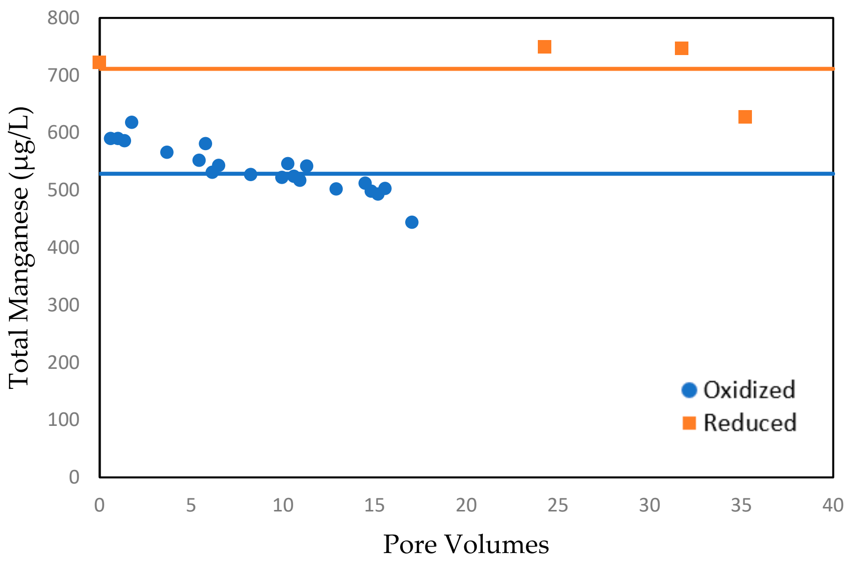

Figure S3. Total Manganese of the column effluent per pore volumes using South River sediment from RRM3.5 under oxidized and reduced conditions.

Table S2. Average THg concentration ± one standard of deviation (n=3) in all bank locations of Constitution Park before and after the bank management.

**Sampling locations**	**Pre Bank Management**		**Post Bank Management**	
	Baseline Conc.(ng/L)	Drainage Conc. (ng/L)	Baseline Conc. (ng/L)	Drainage Conc. (ng/L)
L0			22±10	25±3
L1	84±15	27±7	12±6	30±13
L3	306±142	29±4	15±5	73±33
L5	2215±467	6359±1729	13±6	22±11

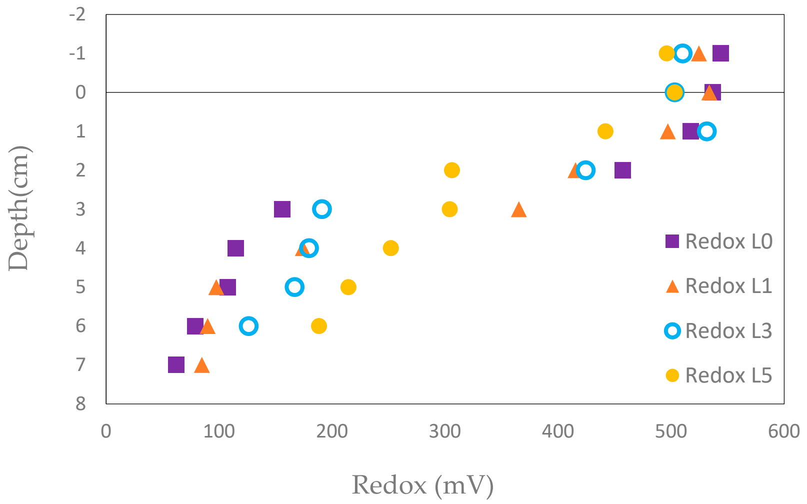

Figure S4. Redox Potential during Drainage condition in 2018 in bank locations of Constitution Park.

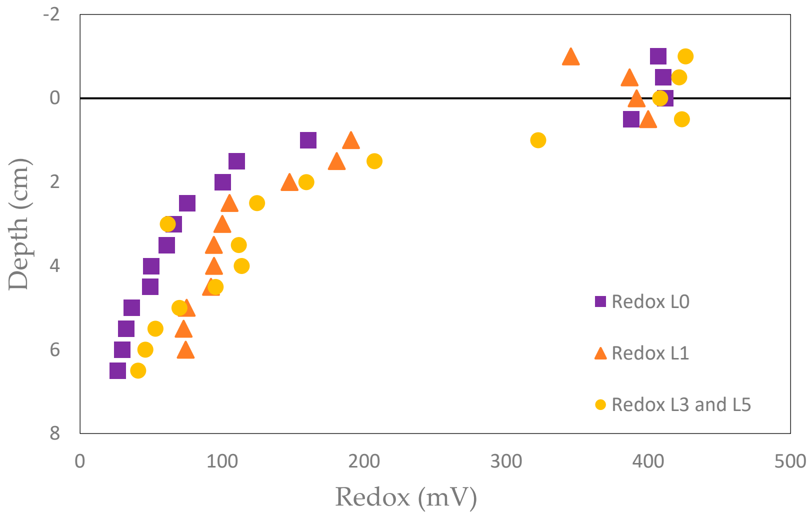

Figure S5. Redox Potential during baseline condition in 2020 in bank locations of Constitution Park. The location 3 and location 5 were both rocky so the point that was selected was between these two locations.

Table S3. Bromide mass-transfer coefficients and estimates of interstitial water velocity from equilibrium diffusion samplers.

Location – Sampling year	Depth below sediment-water interface (cm)	Measured mass-transfer coefficient, k, (cm/d)	Estimate of average pore water velocity, u, (cm/d)
L0, 2018 (Drainage)	20	3.05	> 100
	30	0.46	1
	40	0.27	0
L0, 2020 (Baseline)	10	1.82	39
	20	0.53	1
	30	0.84	4
L1, 2018 (Drainage)	15	0.82	4
	25	0.46	1
L3, 2018 (Drainage)	5	0.69	3
	15	0.69	3
	25	0.54	1
L3, 2020 (Baseline)	7	1.25	12
	16	1.33	15
	24	0.45	1
L5, 2018 (Drainage)	9	1.09	11
	19	1.77	43
	29	3.53	> 100
L5, 2020 (Baseline)	14	3.89	> 100
	24	5.93	> 100
	34	1.20	11
